# Embodied Mindfulness Through Movement: A Scoping Review of Dance-Based Interventions for Mental Well-Being in Recreational Populations

**DOI:** 10.3390/healthcare13172230

**Published:** 2025-09-05

**Authors:** Aglaia Zafeiroudi, Ioannis Tsartsapakis, Ioannis Trigonis, Olga Kouli, Dimitrios Goulimaris, Charilaos Kouthouris

**Affiliations:** 1 Department of Physical Education & Sport Science, University of Thessaly, 42100 Trikala, Greece; kouthouris@uth.gr; 2 Department of Physical Education & Sport Science, Aristotle University of Thessaloniki, 62122 Serres, Greece; ioantsar@phed-sr.auth.gr; 3 Department of Physical Education & Sport Science, Democritus University of Thrace, 69100 Komotini, Greece; itrigon@phyed.duth.gr (I.T.); okouli@phyed.duth.gr (O.K.); dgoulima@phyed.duth.gr (D.G.)

**Keywords:** community-based practices, somatic awareness, embodied learning, contemplative pedagogy, psychological well-being, community health promotion

## Abstract

**Background/Objectives:** Mindfulness has expanded from seated meditation to include embodied practices emphasizing somatic awareness and emotional regulation. Dance offers a creative, accessible pathway to mindfulness, especially in non-clinical settings where movement-based approaches may better support self-regulation, interoception, and well-being. This scoping review investigated empirical studies on dance-based mindfulness interventions targeting non-clinical, amateur and recreational populations. **Methods:** Six databases (PubMed, PsycINFO, Scopus, ERIC, Web of Science, and Google Scholar) were searched for peer-reviewed studies published between 2010 and 2025. Eligible studies combined dance with mindfulness and somatic movement practices and were conducted with non-professional participants of all ages in non-clinical settings. Study selection, data extraction, and appraisal followed PRISMA-ScR guidelines. **Results:** Ten empirical studies met the inclusion criteria, spanning diverse populations from primary school children to older adults. Interventions included Dance/Movement Therapy, ballet with yoga, Sufi-inspired group dance, and school- or community-based mindful movement programs. Reported outcomes included improvements in body awareness, emotional regulation, stress reduction, self-compassion, social connection, and overall well-being. A thematic synthesis identified five domains: (1) psychological and emotional outcomes, (2) embodiment and self-compassion, (3) relational and social benefits, (4) feasibility and acceptability, and (5) sustained and preventive effects. **Conclusions:** Dance-based mindfulness interventions in recreational contexts show promising psychosomatic and emotional benefits. Although the current empirical base is limited and methodologically diverse, this scoping review provides a necessary foundation for understanding this emerging field. There remains a strong need for interventions that are theoretically grounded, culturally sensitive, and pedagogically integrated, particularly within classroom-based dance educational contexts.

## 1. Introduction

Mindfulness, originally rooted in Buddhist contemplative traditions, was developed in monasteries as a meditative discipline oriented toward spiritual liberation and ethical transformation [1,2,3,4]. Within the Buddhist tradition, mindfulness practice has historically been situated within a broader religious and philosophical framework that foregrounds compassion, detachment from craving, and the pursuit of transcendence. This orientation has been sustained through its continuation in meditation retreats and in communal or holistic settings [4].

During the second half of the twentieth century, mindfulness entered Western societies through cultural and social movements that promoted meditation both as a path of spiritual awareness and as a secular resource for well-being [4,5]. A key conduit for this translation was Kabat-Zinn’s Mindfulness-Based Stress Reduction (MBSR), developed at the University of Massachusetts Medical Center in the late 1970s, which enabled the reframing of mindfulness in secular, health-related terms and its systematic study [4,6]. Mindfulness-Based Stress Reduction (MBSR) was established as a secular clinical program designed to integrate mindfulness meditation and yoga into mainstream healthcare [7]. Initially aimed at patients with chronic pain and stress-related disorders, its success facilitated the transfer of mindfulness practice beyond medicine into psychology, education, and community settings. MBSR subsequently became the prototype for a wider range of mindfulness-based interventions (MBCT, MBRP), illustrating how the original program was adapted across diverse domains while maintaining its grounding in contemplative practice [4].

Over subsequent decades, mindfulness has been researched and applied across multiple domains, including clinical medicine and mental health, education, work-place programs, and community/holistic practice, reflecting its broad uptake beyond clinical science. Today, within psychology and the health sciences, it is commonly defined as purposeful, present-moment awareness with a non-judgmental stance [5,8,9,10], with accumulating evidence from randomized trials and reviews indicating benefits for stress reduction, emotional regulation, and overall well-being.

Although most research emphasizes seated, clinical practices, newer approaches advocate for broader, embodied formats of mindfulness. These include walking meditation, mindful walking in clinical and community settings [11], Buddhist walking meditation [12] and psychoballet interventions [13], which also use intentional movement as a vehicle for awareness. Within this wider family of movement-based practices, dance represents a particularly creative, accessible, and socially engaging format for cultivating mindfulness, especially in non-clinical settings [5,9].

Dance offers a creative, accessible, and socially engaging way to cultivate awareness, especially in non-clinical settings. Studies consistently link mindfulness with improved attention, emotional regulation, and reduced psychological distress [8,14,15]. It also supports non-reactivity and reduces rumination and experiential avoidance [16]. Second-generation mindfulness models (SG-MBIs) emphasize ethical, embodied, and sensitive dimensions [17].

Second-generation mindfulness-based interventions (SG-MBIs) were introduced to address limitations of the first generation, which largely emphasized stress reduction and clinical symptom relief. SG-MBIs re-integrate ethical, spiritual, and existential dimensions drawn from Buddhist contemplative traditions, while also engaging contemporary fields such as positive psychology and transpersonal psychology [17]. Their paradigm emphasizes compassion, embodied awareness, and sensitivity to context, thereby broadening the scope of mindfulness beyond clinical symptom management toward holistic well-being [17].

Dance-based mindfulness interventions remain understudied, particularly outside clinical contexts. Given its cultural resonance and ease of access, dance may promote mental well-being through movement, creativity, and interpersonal connection. Although a variety of embodied practices such as yoga [18], tai chi [19], and psychoballet [13] can foster mindful awareness, dance holds distinct characteristics that justify focused exploration. Beyond structured movement or exercise, dance combines creativity, self-expression, and cultural resonance with strong interpersonal and collective elements, which appear to promote greater engagement and commitment [20,21]. Additionally, dance fosters embodied cognition through rhythmic coordination and expressive flow, mechanisms not equally emphasized in other mind–body practices [22]. These qualities allow dance to operate simultaneously as an accessible recreational activity, an expressive art form, and a relational practice, making it a particularly relevant vehicle for integrating mindfulness principles.

This review maps such dance-based mindfulness interventions targeting non-professionals, responding to interdisciplinary calls for inclusive, movement-based approaches to public mental health.

### Background

Dance constitutes a multisystemic, embodied practice with significant implications for emotional, cognitive, and interpersonal functioning, especially when situated within therapeutic frameworks such as Dance Movement Therapy (DMT) [14,15,23,24]. Dance as a structured therapeutic modality is represented by Dance/Movement Therapy (DMT), which emerged in the mid-20th century as part of the creative arts therapies’ movement [25]. Rooted in the pioneering work of Marian Chace in psychiatric settings during the 1940s, DMT has since developed into an established psychotherapeutic approach that emphasizes mechanisms such as mirroring, attunement, and symbolic expression [26]. While DMT was initially applied in clinical contexts, its scope has gradually expanded into community, educational, and wellness settings, where it supports emotional regulation, embodied awareness, and social connection beyond strictly medicalized frameworks [25,26,27]. While Dance/Movement Therapy (DMT) is formally established as a psychotherapeutic modality, scholarship highlights that some of its core mechanisms, such as attunement, kinesthetic empathy, and embodied awareness, are not exclusive to clinical settings and can also inform recreational or community-based dance practices [25,27]. This broader development highlights that DMT, although grounded in psychotherapy, is not confined to clinical populations alone. In parallel, other recreational and community-based dance practices, including conscious dance, improvisational movement, and fitness dance, offer accessible, non-clinical opportunities for cultivating mindful awareness.

Meta-analytic findings indicated that DMT provides medium effect sizes in improving psychological outcomes such as depression, anxiety, and quality of life, particularly within clinical populations, while dance interventions themselves have a larger, albeit more heterogeneous, impact on psychomotor and cognitive skills in broader preventive contexts [27]. DMT leverages movement as a psychotherapeutic medium, enhancing emotional regulation, social integration, and self-awareness through mechanisms including mirroring, non-verbal expression, and affective attunement.

Beyond clinical settings, mindfulness-based approaches have been integrated into a variety of recreational and leisure activities. Practices such as mindful walking, hiking, yoga, and even crafting have demonstrated that everyday movement or creative engagement can become vehicles for cultivating present-moment awareness, non-judgmental observation, and acceptance [11,28,29]. In these contexts, specific mindfulness techniques, including breath awareness, body scanning, intentional movement, and reflective pauses, are adapted to enhance experiential depth and psychological flexibility. Dance represents a particularly salient case within this spectrum: unlike conventional recreational dance, which emphasizes fitness, entertainment, or performance, dance-based mindfulness interventions explicitly integrate meditative attentional strategies with embodied movement. This intentional incorporation of mindfulness distinguishes such interventions from ordinary dance activities, positioning them as hybrid practices that cultivate both somatic awareness and psychosocial well-being [30,31,32].

In parallel, community and performance-based dance contexts have adopted mindfulness and acceptance-based techniques to enhance dancers’ psychological flexibility and attentional focus, although integration into routine practice remains a challenge [33]. Furthermore, recreational and expressive dance has been shown to promote affective connection and embodied self-reflection, offering accessible opportunities for non-clinical populations to engage in experiential self-regulation [34].

Within this embodied paradigm, dance constitutes a dynamic and accessible modality for cultivating mindfulness, particularly in non-clinical and recreational contexts. Evidence from interventions sometimes described as contemplative or mindful movement practices, and more specifically as dance-based mindfulness interventions when dance is the core modality, indicates benefits such as enhanced emotional regulation, attentional anchoring to interoceptive signals, and increased cognitive flexibility [27,34,35,36]. Rather than relying solely on top-down cognitive monitoring, these movement-based formats engage sensorimotor and affective pathways, offering alternative routes for developing mindful awareness, especially among populations for whom verbal or sedentary practices may be less accessible or appealing [37,38].

Accumulating evidence emphasizes the efficacy of mindfulness-informed dance interventions in enhancing interoceptive awareness, psychological flexibility, and emotional integration across diverse populations [14,15,36,37]. These embodied practices appear particularly effective in fostering self-regulation, somatic awareness, and prosocial emotional states, combining the attentional components of mindfulness with the expressive and relational dimensions of movement-based engagement.

Evidence increasingly supports the integration of dance-based mindfulness interventions during childhood and adolescence, highlighting the developmental advantages of fostering embodied self-awareness early in life. Engaging children in creative dance and playful mindful movement facilitates emotion regulation, social bonding, and cognitive flexibility more effectively than purely sedentary or verbal mindfulness practices [39,40]. Moreover, creative dance approaches in educational settings have demonstrated positive effects on self-esteem, resilience, and social connectedness, underscoring the value of introducing these interventions during formative developmental stages.

In adolescent populations, school-based programs such as “Just in TIME” and “The Dance Project” have demonstrated short-term improvements in stress reduction, emotional regulation, and body awareness, suggesting the feasibility of integrating movement-based mindfulness practices within educational settings [41]. These interventions emphasize somatic presence and emotional literacy through structured, mindful movement tasks, elements that are especially supportive in developmental stages characterized by high affective volatility.

Similarly, in performance, the Mindfulness-Acceptance-Commitment (MAC) intervention applied to professional ballet dancers revealed high subjective acceptance and qualitative improvements in attentional control, focus during rehearsals, and reduced performance-related distress, even in the absence of statistically significant quantitative shifts [33]. These findings indicate that mindfulness can be effectively adapted to movement-intensive, high-demand environments where traditional seated meditation may be impractical.

These practical outcomes align with embodiment theory, which posits that emotional and cognitive processes are not exclusively cerebral but grounded in sensorimotor systems [38]. Through rhythmic sequencing and kinesthetic engagement, dance facilitates bottom-up modulation of affective states, activating neural and autonomic pathways associated with emotional regulation, embodiment, and somatic integration [14,17,27]. This embodied access route aligns with theories of interception and sensorimotor awareness, making it particularly relevant for individuals who may find verbal, introspective, or stationary mindfulness approaches less intuitive or effective [15,42]. Accordingly, dance-based mindfulness offers an inclusive, experiential alternative that combines psychological depth with sensory immediacy, supporting mental well-being through active movement and emotional attunement [8,36].

Furthermore, conscious dance forms such as 5Rhythms, Ecstatic Dance, and Open Floor, non-evaluative, improvisational movement practices, have gained traction as avenues for enhancing presence, authenticity, and somatic integration in community settings [43]. Participants in such practices often report increased trait mindfulness, psychological flow, and life satisfaction. Unlike psychotherapeutic interventions, these formats typically occur in inclusive, socially resonant environments, broadening access to body-centered mental health resources.

Despite growing theoretical interest in the neurocognitive mechanisms that might underlie the synergy between dance and mindfulness, such as the “musical pleasure cycle” involving anticipatory-reward feedback loops [23] or predictive coding models of sensorimotor pleasure [42], direct empirical substantiation within the context of recreational or mindfulness-based dance remains limited. While these models offer compelling frameworks suggesting that rhythmic movement and synchronous group participation may activate reward-related neural circuits, current studies are largely speculative or rely on extrapolations from adjacent fields such as music neuroscience and embodied cognition.

Similarly, works by Pickard [24] and Zafeiroudi and Kouthouris [44], while referenced in the discourse around somatic education and mindful movement, were either not accessible or lacked specific empirical application to recreational dance in non-clinical populations. As such, although the conceptual grounding is sound, caution is warranted in generalizing these theoretical propositions. There remains a significant gap in experimental or mixed-methods research to validate these models in applied settings.

In parallel, the sociocultural relevance of dance-based mindfulness practices, has been highlighted in anthropological literature as a response to structural inequities and culturally situated experiences of distress [5]. Within marginalized or trauma-affected populations, embodied practices offer non-verbal, participatory, and context-sensitive avenues for emotional regulation, relational healing, and self-reclamation.

Movement-based interventions, such as dance-based mindfulness interventions, are perceived not merely as therapeutic tools but as socially embedded practices that restore agency and resonance through culturally meaningful modalities. This perspective aligns with somatic education paradigms that emphasize the role of bodily awareness, relational attunement, and non-pathologizing frameworks in fostering psychological resilience [5,14,42]. As such, mindful dance holds promise not only at the level of individual well-being but also as a strategy for community-based mental health support, particularly when adapted to diverse sociocultural realities [15,17].

In response to the increasing demand for inclusive, somatically grounded mental health strategies, this scoping review systematically synthesizes current empirical and theoretical developments on dance-based mindfulness interventions in recreational and community-based contexts. Drawing from evidence in both clinical and non-clinical settings, including Dance Movement Therapy and mindfulness-infused dance programs, the review critically maps intervention modalities, outcome domains, and underlying mechanisms such as interoceptive awareness, psychological flexibility, emotional regulation, and attentional control [27,33,36].

In line with existing literature [27,45,46,47], dance-based mindfulness interventions are defined in this review as structured programs that explicitly integrate mindfulness principles such as present-moment awareness, non-judgmental attention, breathwork and compassion with dance or expressive movement as the primary modality. Koch et al. [27] describe such approaches as integrating expressive dance qualities with mindful awareness techniques, highlighting how movement itself becomes the medium of contemplative practice. Similarly, Wang et al. [47] define dance-based interventions as “movement-with-music activities” (e.g., tango, salsa, ballroom, jazz) that are inherently dynamic, rhythmic, and relational, thereby distinguishing them from static mind–body practices such as yoga or meditation, which focus on posture and breath regulation. Compared to somatic practices such as Pilates, Gyrokinesis, yoga, tai chi, qigong which primarily emphasize alignment, postural control, and internal bodily awareness, dance-based mindfulness interventions add a dimension of expressivity, improvisation, and creative flow without as much energy and interaction as dances performed to music [47]. They also frequently involve interpersonal attunement, group synchrony, and social interaction, which extend the scope of mindfulness beyond the individual body to include relational embodiment.

This review also considers the potential of dance as a culturally responsive, low-barrier modality for populations underrepresented in traditional mental health services. By integrating perspectives from embodiment theory, mindfulness-based intervention research, and community-centered approaches, it contributes to the development of inclusive, scalable programs that support somatic integration, relational healing, and psychological well-being. The findings aim to inform future interventions that extend beyond clinical contexts and respond to growing public health needs for accessible, movement-based mental health strategies.

The purpose of this scoping review is to systematically map and explore existing dance-based mindfulness interventions designed for non-professional, amateur, or recreational participants. Specifically, the review aims to examine how mindfulness principles are integrated into dance practices, and what experiential and psychological outcomes are reported in the literature. This scoping review does not seek to evaluate the quality or effectiveness of interventions but rather to provide an overview of current approaches and identify gaps in knowledge to inform future intervention design. The review addresses the following objectives:

(a) To identify published dance-based mindfulness programs targeting non-professional, non-clinical or recreational participants.
(b) To summarize reported outcomes (psychological, emotional, social, etc.) associated with these programs.
(c) To describe the key characteristics of such interventions (such as country, duration, format, delivery mode, theoretical grounding).
(d) To highlight gaps in the current literature that future research and practice should address.

## 2. Materials and Methods

### 2.1. Study Design

The present study used a scoping review methodology following the foundational framework of Arksey and O’Malley [48], as subsequently refined by Levac et al. [49]. These frameworks provide the methodological foundation for identifying, selecting, and charting evidence. Reporting was further guided by the PRISMA-ScR extension [50]. This combined approach is particularly suited for systematically mapping evidence in emerging, conceptually diverse fields, such as dance-based mindfulness interventions for non-professional populations, where research remains heterogeneous and underdeveloped.

The review was conducted through five key stages:

1. Identifying the research question, focusing on the characteristics, outcomes, and frameworks of existing dance-based mindfulness interventions for non-professional and non-clinical populations.
2. Identifying relevant studies, through a structured database search across PubMed, PsycINFO, ERIC, Web of Science and Scopus using a combination of terms related to mindfulness, dance, and non-professional or recreational participants. In addition, Google Scholar was used as a supplementary search tool to capture grey literature and studies not indexed in the major databases.
3. Study selection, based on predetermined inclusion and exclusion criteria, focusing on empirical studies describing or evaluating dance practices clearly informed by mindfulness interventions for non-professional populations.
4. Charting the data, using a standardized form capturing details about study design, intervention characteristics, population, outcomes, and theoretical basis.
5. Collating, summarizing, and reporting the results, via a narrative synthesis of key themes, trends, and gaps in the literature.

While this scoping review does not aim to assess the effectiveness of interventions through statistical synthesis, it offers a structured overview of the current landscape in the field. The epistemological orientation of the study is informed by constructivist and interpretivist paradigms, acknowledging the subjective, contextual, and experiential nature of both mindfulness and dance [51,52,53]. These perspectives supported the interpretation of findings across diverse participant experiences and program structures. The review protocol was prospectively registered in PROSPERO (CRD420251054099).

### 2.2. Inclusion and Exclusion Criteria

The inclusion criteria were developed to ensure the selection of relevant, methodologically sound, and context-appropriate studies for this scoping review [49,54]. Studies were eligible for inclusion if they met the following criteria:

Publication Period and Language: Peer-reviewed articles published in English between 2010 and 2025.

Population: Children, adolescents, adults participating as non-professional, amateur, recreational dancers. Professional or elite dancers were excluded to ensure focus on general populations engaging in dance primarily for wellness or leisure purposes.

Intervention: The study described or evaluated an intervention integrating mindfulness components (awareness, non-judgment, acceptance) with dance-based practices. Both formal (for example structured MBSR-inspired sessions) and informal mindfulness approaches (for example guided body awareness) were considered. DMT programs are also considered only when implemented in non-clinical settings (e.g., schools, communities).

Setting: Interventions conducted in non-clinical settings such as community centers, schools, universities, wellness programs, or informal group contexts. Clinical populations or therapeutic treatments in healthcare settings were excluded.

Study Design: Any empirical study design was eligible, including qualitative, quantitative, and mixed-methods approaches. The aim was to capture a comprehensive and diverse evidence base.

By focusing on dance-based mindfulness interventions designed for non-professionals in real-world, non-clinical settings, the review prioritized studies with broader public health relevance and applicability to community-based wellness initiatives.

Studies were excluded from the review if they met any of the following conditions:

Population-Based Exclusion: Studies that exclusively included professional dancers, elite performers, competitive dancers or participants with clinically diagnosed mental or physical disorders (e.g., psychiatric, neurological, or chronic medical conditions).

Setting-Related Exclusion: Interventions implemented within clinical, hospital-based, therapeutic, or psychotherapeutic environments were excluded.

Lack of Dual Intervention Focus: Studies were excluded if they did not clearly integrate both mindfulness-based components (for example attentional focus, body awareness, non-judgment, acceptance) and movement-based or dance-based practices. Interventions that included only general movement or general fitness modalities or only mindfulness or solely on somatic movement without dance elements were not considered.

Non-Empirical or Inaccessible Sources: Studies were excluded if the full text was not accessible, if the methodology was vague or incomplete, or if the publication was in a language other than English.

Lack of Structured Intervention or Outcomes: Studies were excluded if they mentioned mindfulness or dance but did not describe a clearly structured intervention protocol, did not identify specific experiential or psychological outcomes, or failed to provide relevant data for extraction aligned with the review’s objectives.

### 2.3. Search Strategy

Studies were sourced from well-established academic databases including PubMed, Scopus, PsycINFO, ERIC, and Web of Science. In addition, Google Scholar was used as a supplementary search tool to capture grey literature and studies not indexed in the major databases. Given the large number of results typically generated in Google Scholar, a manual search strategy was applied. The first 100 results per keyword combination were screened by title and abstract, ordered by relevance [55]. These databases were selected for their breadth of coverage in psychology, health sciences, and education, which are highly relevant domains for dance-based mindfulness interventions. This multi-source approach is consistent with best practices in evidence synthesis for qualitative and mixed-methods reviews [56,57].

The search strategy was informed by the SPIDER framework (Sample, Phenomenon of Interest, Design, Evaluation, Research type), which is commonly applied in qualitative and mixed-methods reviews [58]. The primary focus was on psychological and mental health outcomes (e.g., stress reduction, emotional regulation, self-awareness). However, when studies additionally reported physical (e.g., balance, flexibility) or social outcomes (e.g., connectedness, group belonging), these were also extracted and summarized to provide a comprehensive mapping of intervention effects. A detailed summary of the SPIDER framework is presented in Table 1.

Boolean operators and keyword combinations were applied across databases. A sample search string was: (“mindfulness” OR “meditation” OR “awareness” OR “contemplative practice” OR “DMT” OR “MBSR” OR “MAC approach”) AND (“dance” OR “movement” OR “creative movement” OR “expressive movement” OR “embodied”) AND (“intervention” OR “protocol” OR “program”) AND (“recreational” OR “non-professional” OR “community” OR “amateur” OR “non-clinical”). In Google Scholar, the same keyword combinations were applied with filters for year of publication (2010–2025) and English-language studies (Table 2).

### 2.4. Study Selection Process

**Figure 1:**
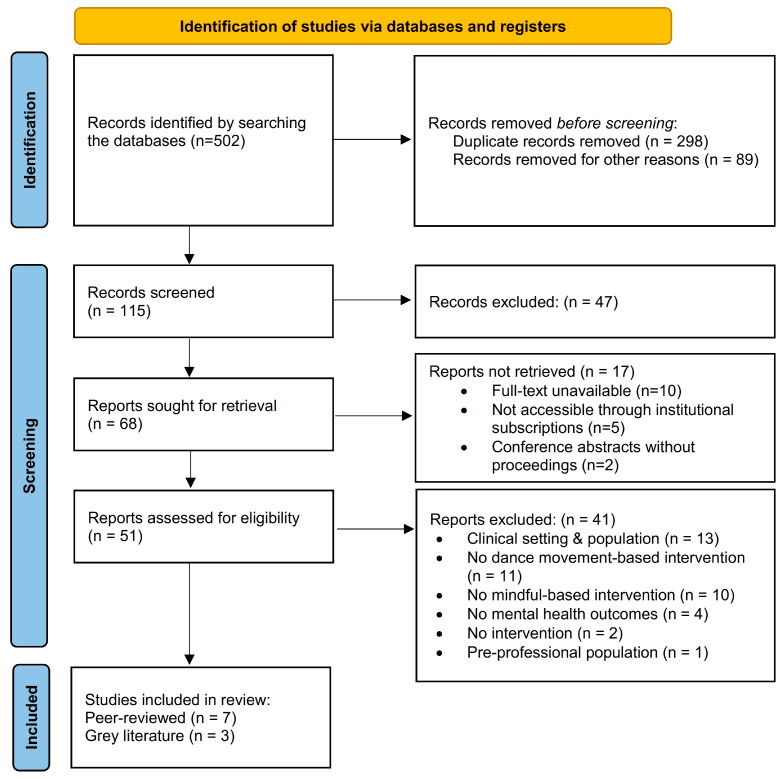
PRISMA 2020 flow chart.

The study selection process followed the Preferred Reporting Items for Systematic Reviews and Meta-Analyses (PRISMA 2020) guidelines [50,59]. A total of 502 records were identified through database searches. After removing 298 duplicates and 89 records for other reasons, 115 records remained for screening. During the title and abstract screening, 47 records were excluded. The remaining 68 reports were sought for full-text retrieval; however, 17 could not be obtained (10 full texts unavailable, 5 inaccessible due to institutional subscription restrictions, and 2 conference abstracts without proceedings). In total, 51 full-text reports were assessed for eligibility. Following full-text evaluation, 41 reports were excluded for the following reasons: conducted in clinical settings or involving clinical populations (*n* = 13), no dance movement-based intervention (*n* = 11), no mindfulness-based component (*n* = 10), no mental health outcomes (*n* = 4), no intervention protocol (*n* = 2), and pre-professional participants (*n* = 1). Ultimately, 10 studies were included in the final synthesis, comprising 7 peer-reviewed articles and 3 grey literature sources. A visual representation of the study selection process is provided in the PRISMA 2020 flow diagram (Figure 1).

## 3. Results

A total of ten empirical studies published between 2010 and 2025 were included. Of the 10 included studies, 7 were peer-reviewed and 3 were grey literature sources, reflecting the emerging and exploratory nature of the field. Studies were conducted across Sweden, the USA, Spain, and Australia, reflecting diverse cultural and educational contexts. Research designs varied and comprised randomized controlled trials [36,60], quasi-experimental designs [61], mixed-methods studies [62,63,64], and qualitative case studies [32,65] (Table 1).

Sample sizes ranged from a single adolescent participant [65] to 133 primary school children [66]. The studies involved adolescents and young adults, particularly high school and university students, although adults and especially older adults were also represented. Populations included general community groups, student cohorts, and individuals with self-reported psychosomatic symptoms, stress, depression, or mild cognitive decline, but were not conducted in clinical contexts. Overall, the included studies primarily targeted non-clinical and community-based participants, rather than professional or competitive dancers or clinical patient populations. (Table 3).

The interventions encompassed a wide range of dance-based mindfulness interventions, including mindful movement approaches. Several studies employed Dance/Movement Therapy (DMT) frameworks, either in structured academic settings [36] or as part of wellness curricula [65]. Other interventions integrated specific dance forms, including, ballet combined with yoga techniques [62], and African, jazz, or contemporary dance with improvisation [60]. Sufi-inspired mindful dance protocols were implemented in group-based university contexts [61], while school-based programs incorporated a variety of dance styles with mindfulness components into the curriculum [41,64,66].

Mindfulness techniques embedded in the interventions included breath awareness, body scans, relaxation exercises, movement-based reflection, non-judgmental attention, and compassion-focused practices. Some programs emphasized social synchrony and group reflection [61,63], while others highlighted embodied attentional focus and improvisational expression [60]. Intervention length varied from short-term programs of five to seven weeks [61,62] to longer-term curricula lasting up to eight months [60] or spanning a full school year [66]. Delivery formats included in-person classes, community-based workshops, and, more recently, online platforms for older adults [63].

Across the included studies, outcomes were reported in psychological, physical, social, and behavioral domains. While most studies focused primarily on psychological and emotional outcomes, a subset also reported physical (e.g., balance, coordination) and social outcomes (e.g., connectedness, group belonging), whereas others did not assess these dimensions explicitly. Among adolescents, interventions were associated with reductions in stress, psychosomatic symptoms, and tiredness, as well as improvements in alertness, self-rated health, and emotional regulation [41,60,64]. School-based programs further demonstrated gains in executive functioning, internalizing and externalizing behaviors, and motor coordination [66].

In adult populations, dance-based mindfulness interventions contributed to reductions in depression, stress, and bodily dissociation, while enhancing mindfulness, self-compassion, and body awareness [32,36]. Additional benefits included improvements in life satisfaction, emotional expression, and problem-solving ability. University-level programs also reported increases in compassion and social connectedness, mediated through shared flow and group synchrony [61].

For older adults, online dance-based mindfulness programs drawing from DMT frameworks (M-DMT) reduced loneliness and depression while supporting positive affect, psychological well-being, and social engagement [63]. Qualitative findings across studies consistently highlighted enhanced body awareness, emotional regulation, creativity, and empowerment, as well as improved interpersonal connection. Overall, despite heterogeneity in populations, modalities, and assessment tools, outcomes were consistently positive, indicating psychosomatic, emotional, and relational benefits of dance-based mindfulness interventions in non-clinical settings.

Feasibility and acceptability outcomes were consistently positive across the included studies. High recruitment and retention rates were reported in school-based programs, with participation exceeding 90% in some cases [66]. Adherence was also strong in long-term interventions, such as Duberg et al. [60], where 67% of participants attended at least half of the sessions. No adverse events were reported in any study. All included studies reported obtaining ethical approval, ensuring adherence to established standards of research integrity and participant protection.

Participants frequently described the interventions as enjoyable, engaging, and meaningful. Qualitative feedback highlighted themes of safety, empowerment, and personal growth [32,64]. Teachers and parents reported high levels of integration into educational settings, with positive perceptions of student engagement and curriculum compatibility [65,66]. In community-based interventions, participants expressed preference for embodied and creative elements, which were perceived as supportive for self-awareness and emotional regulation.

The adaptability of delivery formats was also evident. Programs were successfully implemented across schools, universities, and community contexts, while online formats for older adults demonstrated feasibility and accessibility in geographically diverse regions [63]. Collectively, these findings suggest that dance-based mindfulness interventions are acceptable and practicable across a range of populations, age groups, and settings. Table 4 provides a comprehensive summary of the studies included in this review.

To better understand the mechanisms through which dance-based mindfulness interventions exert their impact, the included studies were analyzed thematically. Five overarching categories emerged:

1. Psychological and Emotional Outcomes: Across age groups and contexts, dance-based mindfulness interventions were linked with improvements in psychological well-being, emotional regulation, and stress management. For adolescents, participation reported reductions in stress, psychosomatic symptoms, and tiredness, as well as improvements in self-rated health, emotional regulation, and overall vitality [41,60,64]. In university students and young adults, interventions fostered significant decreases in perceived stress and depression, alongside enhanced mindfulness, compassion, and life satisfaction [36,61,62]. Among older adults with mild cognitive decline, online mindfulness-based dance/movement therapy (M-DMT) resulted in reductions in loneliness and depressive symptoms, coupled with gains in positive affect and overall well-being [63]. These findings highlight the potential of such interventions to promote emotional resilience and psychological health across the lifespan.
2. Embodiment and Self-Compassion: A central mechanism emerging from the studies was embodied awareness, the integration of body and mind through mindful movement practices. Techniques such as breathwork, body scans, improvisational dance, and reflective exercises consistently enhanced interoceptive awareness, self-compassion, and trauma processing. For example, Dancing Mindfulness facilitated improvements in body awareness and emotional expression among trauma-exposed participants, promoting safety and empowerment [32]. Similarly, Rodríguez-Jiménez et al. [36] demonstrated that DMT-based interventions significantly increased body awareness, decreased bodily dissociation, and improved life satisfaction and well-being in university students. Mindful ballet-yoga combinations further reduced performance anxiety and enhanced attentional focus [62], while school-based programs integrating relaxation and non-competitive enjoyment promoted embodied presence and acceptance among adolescents [41]. These findings highlight embodiment as a foundational pathway to psychological regulation and self-compassion.
3. Relational and Social Benefits: Dance-based mindfulness programs also fostered social and relational benefits, emphasizing co-regulation and collective experience. University-based mindful dancing protocols grounded in Sufi principles enhanced compassion for others, mediated by shared flow and perceived emotional synchrony within group contexts [61]. In school-based interventions, programs fostered non-competitive enjoyment, peer inclusion, and collaborative creativity [64,66]. Among older adults, the online M-DMT program supported stronger social engagement and a sense of belonging despite geographical distance, demonstrating the adaptability of digital platforms for relational outcomes [63]. These findings suggest that mindfulness-based dance creates opportunities for connection, trust-building, and social cohesion.
4. Feasibility and Acceptability: Across studies, interventions demonstrated high feasibility, engagement, and acceptability. School-based programs reported exceptional recruitment (up to 91%) and retention (up to 98%), with teachers noting successful integration into curricula and high student engagement [64,66]. Community-based initiatives similarly achieved strong commitment and positive participant experiences, with Duberg et al. [60] reporting that 67% of adolescents attended at least half the sessions and 91% rated the experience positively. Qualitative feedback highlighted feelings of safety, empowerment, and enjoyment as central to sustained participation [32]. Online formats were also feasible and well-received among older adults, suggesting that digital delivery can expand accessibility while maintaining participant engagement [63].
5. Sustained and Preventive Effects: Although limited in number, several studies indicated long-term and preventive benefits. In adolescents, improvements in self-rated health were sustained up to 12 months after intervention completion [60], and reductions in daytime tiredness and gains in alertness persisted at 20-month follow-up assessments [41]. Among university students, mindfulness-based DMT buffered against stress increases during exam periods, suggesting a preventive role in high-stress academic contexts [36]. These findings point to the potential of dance-based mindfulness to deliver durable effects. However, the scarcity of long-term data and methodological variability across studies emphasize the need for future research with extended follow-up periods.

The thematic synthesis highlights that dance-based mindfulness interventions provide multifaceted benefits spanning psychological, embodied, social, and behavioral domains. While heterogeneity in populations, modalities, and outcome measures limits direct comparisons, the evidence base collectively supports the integration of mindful movement practices into educational, community, and clinical-preventive frameworks.

While this review did not apply a formal risk of bias tool due to the scoping nature and heterogeneity of the included studies, complementary strategies were employed to enhance transparency and rigor. The Template for Intervention Description and Replication (TIDieR) checklist was used to systematically map intervention characteristics (Appendix A), a thematic synthesis was conducted to identify core domains across studies, and an age-group analysis was performed to illustrate the distribution of participant populations (Appendix B). In addition, studies were categorized by design type, source, and country of implementation to provide an overview of methodological diversity and geographical spread (Table 1).

**Figure 2:**
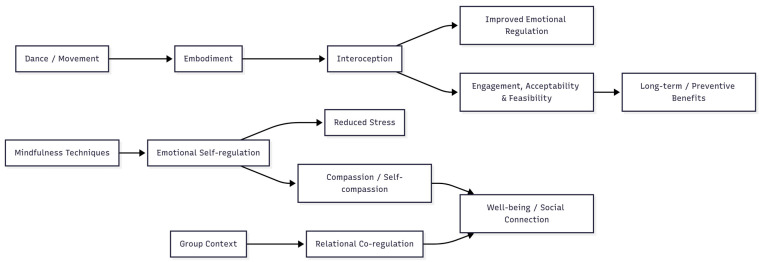
Conceptual model synthesizing core components, mechanisms, and outcomes of dance-based mindfulness interventions identified in the review.

To integrate these findings, a conceptual model was developed (Figure 2) to illustrate the key intervention components, proposed mechanisms of action, and the observed outcomes.

The conceptual model (Figure 2) synthesizes the main findings across the included studies and illustrates the mechanisms through which dance-based mindfulness interventions exert their effects. Dance and movement practices were associated with enhanced embodiment and interoceptive awareness [41,60], which contributed to improved emotional regulation [36,64] and higher engagement, acceptability, and feasibility across school- and community-based contexts [65,66]. Mindfulness techniques integrated into the interventions supported emotional self-regulation, leading to reductions in stress and psychosomatic symptoms [36,61], as well as increased compassion and self-compassion [32,61]. Furthermore, group-based contexts facilitated relational co-regulation, fostering well-being, social connectedness, and shared experiences of flow [61,63]. These mechanisms highlight the effectiveness of dance-based mindfulness interventions by supporting sustained participation and, in several cases, producing long-term and preventive benefits. For example, improvements in self-rated health were maintained up to 12 months post-intervention [60], while reductions in daytime tiredness and increases in alertness persisted up to 20 months [41].

## 4. Discussion

This scoping review systematically mapped ten empirical studies published between 2010 and 2025 that investigated dance-based mindfulness interventions in non-clinical and recreational settings. In line with the predefined objectives (a–d), the synthesis aimed to: (a) identify and describe existing interventions; (b) summarize reported outcomes; (c) analyze key contextual and methodological characteristics; and (d) highlight conceptual boundaries and gaps for future research.

By integrating findings from Sweden, Spain, the USA, and Australia, the review provides an overview of the scope and diversity of this emerging field, which spans populations from school-aged children to older adults. Across studies, mindfulness principles were operationalized through a wide range of dance modalities, including Sufi-inspired collective practices [61], ballet-based movement [62], Dance/Movement Therapy (DMT) frameworks [36], and school-based mindful dance programs [41,60].

However, the limited number of studies identified raises an important question emphasized by peer reviewers: does this scarcity reflect a true research gap or overly restrictive inclusion criteria? The evidence suggests a combination of both factors. On one hand, the integration of dance and mindfulness remains a nascent field compared to well-established frameworks such as Mindfulness-Based Stress Reduction (MBSR) [6] or Mindfulness-Acceptance-Commitment (MAC) approaches [67]. On the other hand, the inclusion criteria specifically excluded studies conducted in clinical populations, interventions lacking explicit mindfulness components, and programs based solely on somatic practices such as yoga or Pilates. While this focus strengthens the conceptual coherence of the review, it also inevitably narrows the available evidence base, resulting in a small number of eligible studies.

Furthermore, the review highlights a recurring terminological inconsistency across the literature. Terms such as “dance-based mindfulness,” “mindfulness-based dance,” “mindful movement,” and “contemplative movement” are often used interchangeably by authors, despite referring to interventions with different emphases. This inconsistency complicates cross-study comparisons and suggests a need for greater conceptual clarity in future research.

While the findings reveal growing academic and applied interest in the intersection of dance, embodiment, and mindfulness, the evidence remains fragmented and methodologically heterogeneous. This highlights the need for further empirical work to establish standardized frameworks while preserving the artistic, embodied, and culturally sensitive dimensions that distinguish dance-based approaches from more conventional mindfulness programs.

### 4.1. Identified Interventions: Scope and Nature

In line with Objective (a), this section identifies and characterizes the dance-based mindfulness interventions reported in the ten included studies. Across non-clinical and recreational contexts, the interventions encompassed a wide spectrum of dance and movement practices, reflecting both methodological diversity and cultural specificity.

Several studies drew upon structured frameworks such as Dance/Movement Therapy (DMT) that integrated improvisational and yoga-based elements [27,36]. Others used culturally rooted practices, including Sufi-inspired collective dances emphasizing emotional synchrony and shared flow [61], as well as programs integrating contemporary, jazz, and African dance traditions in school-based interventions targeting adolescent girls [41]. Additional approaches explored hybrid somatic modalities, such as yoga-infused mindful movement within higher education [36], community-based programs like “Dancing Mindfulness” [32], and mindfulness-informed dance for primary school children [66] or older adults with cognitive decline [63].

Despite these differences, all interventions incorporated core mindfulness elements such as attentional regulation, body awareness, non-judgmental acceptance, and emotional regulation, thereby partially aligning with both first-generation frameworks grounded in Buddhist-inspired meditative practices [6] and second-generation approaches emphasizing embodiment and relational awareness [17,68,69]. However, while mindfulness served as a shared foundation, the dance component varied widely: some programs treated dance as an artistic and aesthetic experience [27], while others framed it primarily as a vehicle for mindful movement and stress reduction [32].

This heterogeneity raises two critical issues. First, the terminological inconsistency observed across studies, where labels such as “dance-based mindfulness,” “mindfulness-based dance,” “mindful movement,” and “contemplative movement” were used interchangeably complicates cross-study comparisons and meta-analyses. Establishing clearer operational definitions is therefore essential for future research. Second, the adaptation of culturally rooted practices (e.g., Sufi-inspired dance or Taiji-based mindful movement) into Western recreational and educational contexts introduces important questions of cultural translation and participant resonance [70]. While these practices may offer symbolically rich frameworks for cultivating mindfulness, their decontextualization risks reducing them to generic “mindfulness techniques,” potentially overlooking their historical meanings and spiritual depth.

Delivery formats varied substantially, ranging from short-term workshops of five weeks [36,61] to multi-month school-based programs lasting six to eight months [41,60]. However, a recurring observation across studies was the limited curricular or structural integration of these interventions. Most were implemented as standalone modules, supplementary projects, or pilot programs, rather than being embedded within sustained pedagogical or community frameworks. This fragmentation may restrict opportunities for participants to internalize and sustain mindfulness-related traits and embodied awareness in everyday educational or community contexts.

Finally, a tension emerges between interventions prioritizing dance-specific pedagogy and those adopting a general movement-based wellness perspective. Programs grounded in expressive dance and improvisation [36,41] engaged more directly with the aesthetic, relational, and affective dimensions of dance, aligning with broader arts-based mindfulness literature that frames dance as a distinctive form of embodied meaning-making [71,72]. In contrast, interventions emphasizing somatic practices (e.g., yoga-based mindful movement) tended to foreground body awareness while downplaying dance as an artform [32,36]. This distinction is conceptually significant, while more generalized mindful movement may enhance accessibility, programs that preserve dance-specific pedagogy are better positioned to leverage the unique expressive and cultural potential of dance.

The findings indicate that dance-based mindfulness interventions occupy an interdisciplinary space at the intersection of arts, embodiment, and contemplative practice, but the field requires greater conceptual clarity, cultural reflexivity, and theoretical grounding to strengthen future research and practice.

### 4.2. Reported Outcomes and Experiential Themes

In line with Objective (b), this review synthesized the outcomes reported across the ten included studies, revealing a consistent pattern of positive experiential, psychological, and social effects of dance-based mindfulness interventions. Despite significant methodological heterogeneity, converging findings suggest improvements in stress regulation, emotional awareness, body-based self-perception, and social connectedness across diverse non-clinical and recreational populations.

Several studies mentioned enhanced emotional regulation, self-compassion, and affective flexibility following participation in dance-based mindfulness programs. For instance, trauma-sensitive interventions such as Dancing Mindfulness [32] were associated with increased self-compassion and improved trauma processing capacity, aligning with trauma-informed frameworks that position movement, rhythm, and embodied expression as critical mechanisms for regulating arousal [73]. Similarly, collective Sufi-inspired dances fostered emotional synchrony, compassion, and shared flow, suggesting that embodied group practices may amplify relational and emotional attunement [61].

From an affective neuroscience perspective, these findings may reflect the role of interoceptive awareness in modulating emotional regulation. Studies have shown that mindful movement activates insula and somatosensory networks associated with body-based awareness and emotion processing [74,75]. By engaging the body in rhythmic and aesthetic movement, dance-based mindfulness may enhance bottom-up pathways linking sensorimotor integration with affective balance, a mechanism less emphasized in purely meditative frameworks like MBSR [6].

Evidence also points to psychosomatic benefits, particularly among adolescents. Duberg et al. [60] and Areskoug Sandberg [41] reported reductions in self-rated stress, daytime fatigue, and psychosomatic symptoms in school-based dance mindfulness programs, with effects sustained up to 12 months post-intervention in some cases. Rodríguez-Jiménez et al. [36] provided a stronger methodological contribution by triangulating psychometric data with biological markers (cortisol levels), demonstrating reductions in bodily dissociation and stress-related biomarkers. This integration of physiological outcomes remains rare across the reviewed literature and should be prioritized in future research.

Comparatively, standardized protocols like MBSR and MAC frequently report similar psychosomatic benefits, but achieve them through prolonged, structured practice and formal meditation training [38,76,77]. By contrast, the reviewed interventions achieved comparable short-term gains through embodied, aesthetic, and socially grounded pathways, suggesting an alternative mechanism of action worth further exploration.

Group-based interventions frequently highlighted improvements in social connection, relational co-regulation, and collective belonging. Shim et al. [63] found reductions in loneliness and depressive symptoms among older adults engaging in online mindful dance programs, while Theroux et al. [66] demonstrated improved behavioral regulation and peer interactions in school-aged children. These findings align with theories of embodied intersubjectivity and social synchrony, which posit that shared rhythmic movement enhances relational attunement and fosters group cohesion [27,78]. Unlike solitary mindfulness practices such as MBSR, which prioritize individual awareness, dance-based approaches may leverage collective embodiment as an active mechanism for psychological well-being.

### 4.3. Key Characteristics and Contextual Dimensions

In line with Objective (c), this review examined the key characteristics and contextual dimensions shaping dance-based mindfulness interventions in non-clinical and recreational settings. Findings indicate that these interventions are deeply influenced by sociocultural contexts, conceptual frameworks, and pedagogical intentions, distinguishing them from more standardized mindfulness programs.

First, unlike MBSR or MAC, which are extensively manualized, standardized, and widely replicated, the reviewed dance-based interventions displayed limited formalization. As highlighted in the TIDieR appraisal (Appendix A), most of the included studies did not provide treatment manuals, fidelity measures, or standardized delivery protocols. This creates challenges for research, as heterogeneity in session length, facilitator expertise, and movement practices reduces replicability and complicates cross-study comparisons. At the same time, this relative lack of standardization can be viewed as a strength in practice: it allows dance-based interventions to remain adaptable to participants’ needs, cultural contexts, and group dynamics, thereby fostering creativity and responsiveness. By contrast, the structured eight-week MBSR format, combining guided meditations, group discussions, and home practice, has enabled consistent implementation and robust testing across diverse populations and contexts [79]. Similarly, MAC protocols in performance psychology have gained traction precisely because their manualized structure ensures fidelity and comparability [80,81,82]. According to all these, while dance-based mindfulness approaches may currently lack the scalability and cumulative evidence base of MBSR and MAC, their openness and flexibility may be integral to their distinctive therapeutic value.

Another key distinction concerns integration into daily life. MBSR and related programs are explicitly designed to extend mindfulness practices beyond the intervention setting, fostering habits that can be sustained over time [79]. By contrast, most dance-based mindfulness interventions identified in this review were short-term, supplementary modules or pilot programs, often detached from participants’ regular educational or community routines. This limited integration may reduce opportunities for embodied mindfulness to become part of daily practice, particularly in school or community contexts.

The conceptual model (Figure 2) provides a concise synthesis of the experiential findings, highlighting interoceptive awareness, emotional self-regulation, and relational co-regulation as central pathways. These mechanisms resonate with but also differ from those emphasized in established mindfulness programs such as MBSR and MAC, where standardization and formalized protocols dominate. By contrast, the reviewed dance-based interventions appear to rely on embodied, relational, and contextually adaptive mechanisms, which may represent both a strength and a challenge for scalability and cross-study comparison.

Finally, from a methodological perspective, the evidence base for dance-based mindfulness interventions in recreational settings remains preliminary. Sample sizes were generally small, reliance on self-report measures was common, and few studies incorporated follow-up assessments or physiological data. This contrasts with the larger-scale, longitudinal evaluations seen in MBSR, MAC, and other established programs.

These comparisons suggest that while dance-based mindfulness interventions share overlapping goals with established mindfulness approaches, such as stress reduction, well-being enhancement, and emotional regulation, they pursue these aims through a distinct experiential and embodied pathway. To strengthen their position within the broader mindfulness landscape, future work will need to enhance theoretical grounding, standardization, and long-term evaluation while retaining the unique artistic and somatic qualities of dance.

The ten included studies illustrate how dance-based mindfulness interventions are deeply shaped by their sociocultural contexts. Research conducted in Northern Europe (Sweden) tended to emphasize collective participation and psychosocial well-being in school or youth settings [41,60], where dance was framed as a vehicle for inclusion, enjoyment, and stress reduction. In Southern Europe (Spain), interventions such as Pizarro et al. [61] and Rodríguez-Jiménez et al. [36] drew on expressive or therapeutic group formats, embedding dance within relational and affective dimensions such as compassion, synchrony, and body awareness. This reflects a biomedical and performance-enhancement discourse, positioning mindfulness not only as an expressive resource but also as a scientifically validated stress-management strategy.

In the United States, a wider spectrum of applications was evident: from therapeutic trauma-informed work [32], to educational and developmental contexts in schools and universities [62,64,65], to older-adult interventions delivered online [63]. This variety mirrors the U.S. tradition of eclectic integration of mindfulness with somatic psychology, dance education, and digital delivery, yet it also raises questions about theoretical coherence and cultural specificity. Similarly, the Australian study [66] was embedded within formal institutional structures, clinical research on tango for depression and school-based mindful movement programs, reflecting a pragmatic focus on feasibility, retention, and measurable psychosocial outcomes.

An examination of both included and excluded studies reveals a distinct geographical distribution. This geographical distribution highlights both the universality and cultural contingency of mindful dance. On one hand, common experiential themes such as shared flow, emotional regulation, and body awareness, emerged across continents, suggesting that dance may naturally support mindfulness through embodied synchrony. On the other hand, the ways in which mindfulness was conceptualized and operationalized varied significantly: in Spain and Sweden, it was linked to social connection and inclusion; in the U.S., to trauma healing, education, and aging; and in Australia, to clinical feasibility and school integration.

A critical limitation is that most studies did not explicitly acknowledge or analyze the cultural origins of the dance forms employed. Practices such as Taiji, or Sufi-inspired dance were adapted into intervention protocols without reflection on issues of cultural translation, appropriation, or participant resonance. This risk reducing culturally embedded symbolically rich practices to decontextualized “mindfulness techniques,” thereby overlooking the cultural and historical dimensions that give these practices meaning. Moreover, few studies examined how participants’ own cultural identities influenced their engagement with the interventions, leaving open the question of whether certain populations experienced these programs as culturally relevant, alienating, or transformative.

Thus, while the reviewed evidence suggests that dance-based mindfulness interventions can generate psychosocial and educational benefits across different societies, there remains a need for cultural reflexivity, theoretical transparency, and community co-design. Future research should more carefully consider the implications of transferring traditional movement practices into new contexts, explicitly address potential issues of appropriation, and develop frameworks that balance methodological rigor with cultural sensitivity. By situating mindfulness not only within generic wellness discourses but also within the symbolic and relational depth of dance traditions, the field can move toward more authentic, inclusive, and contextually grounded practices that respect both the artform and the communities in which it is practiced.

### 4.4. Conceptual Boundaries, Gaps and Limitations

In line with Objective (d), this review also sought to highlight conceptual boundaries, gaps, and limitations in the current literature. Although ten empirical studies were included, several additional works that initially appeared relevant were excluded due to methodological or contextual misalignment with the predefined criteria. For example, Majore-Dusele et al. [83] was excluded because it was conducted in a clinical rehabilitation context and involved patient populations. Similarly, Caldwell et al. [37] and Wallman-Jones et al. [84] explored somatic practices such as Pilates, Taiji Quan, Gyrokinesis, Feldenkrais, and yoga, but did not implement a dance-based intervention. Rice et al. [85] likewise evaluated a mindful movement program for children involving Tai Chi, yoga, biomechanical warm-ups, and imaginative play, but no dance elements. Spaccapanico Proietti et al. [86] investigated a multimodal mindful movement program in young adults, yet again without a dance component. Laird et al. [43], although relevant to embodied awareness, was a cross-sectional survey with no intervention protocol, while de Sousa and Shapiro [68] presented a conceptual discussion of contemplative pedagogy rather than an empirical program. Pinniger et al. [87,88,89,90] were not included, as they do not clearly refer to mindfulness techniques beyond the focus on consciousness of walking, awareness of one’s own and partner’s body, and resistance and transference of weight. Similarly, the study by Liu et al. (2022) [91] compared an MBSR program with a fitness dance group, but did not include any mindfulness components.

Further exclusions from grey literature required consideration. The doctoral thesis by Areskoug Sandberg [41] comprised three distinct studies: of these, only “The Dance Project” was included, while the other two, one involving a clinical population with functional abdominal pain disorders (dance and yoga) and one consisting of a school-based mindfulness program without dance, were excluded. Likewise, Lefebvre Sell [92] was excluded because its sample consisted exclusively of pre-professional dance students in a BA Dance Theatre program, limiting generalizability to broader non-clinical contexts. Finally, the thesis by Sharp Akeila [93] was excluded, as it does not present a clearly structured mindfulness program.

Although excluded, all these works remain theoretically valuable. Many engage with somatic, trauma-sensitive, or contemplative frameworks that emphasize the broader potential of embodied awareness practices. Their exclusion highlights both the definitional ambiguities surrounding what constitutes a “mindfulness-based dance” intervention and the still-developing nature of the field. As emphasized in the introduction, mindfulness and dance are culturally situated and variably defined across disciplines. Some excluded studies equated mindfulness with presence or spiritual awareness without linking these concepts to established constructs such as attentional regulation or cognitive decentering. Others integrated movement for expressive or symbolic purposes without systematically embedding mindfulness techniques. These exclusions illustrate the importance of developing clearer conceptual and methodological boundaries. Future reviews would benefit from frameworks that distinguish between a) Clinical vs. non-clinical populations, b) Therapeutic vs. educational/recreational aims, c) Dance-based vs. general mindful movement approaches, and d) Structured vs. intuitive, arts-based explorations. By clarifying these distinctions, the field can move toward greater theoretical coherence while retaining the richness of interdisciplinary perspectives.

Including grey literature was a deliberate methodological choice in this review. Excluding such sources would have risked overlooking valuable empirical data, particularly on feasibility, acceptability, and innovative program design. While grey literature may lack the same degree of peer-review rigor, it contributes to mapping the full scope of available evidence, mitigates publication bias, and ensures that early but relevant work is captured. This aligns with scoping review methodology, which prioritizes breadth and transparency over critical appraisal, and is especially important in fields where formal publication pathways remain limited.

While interventions such as Dance Movement Therapy (DMT) or Mindfulness-Acceptance-Commitment (MAC) protocols differ conceptually and structurally from programs like Mindfulness-Based Stress Reduction (MBSR), they were included in this review when they clearly integrated dance as a central modality rather than general mindful movement. Unlike MBSR, which is standardized and meditation-focused, or MAC, which targets performance contexts through cognitive reframing, the selected studies employed dance as an embodied, expressive vehicle for cultivating mindfulness. This focus on dance-specific practices, rather than on yoga, Pilates, or other somatic techniques, ensures conceptual alignment with the scope of the review and highlights the unique contributions of dance to the cultivation of mindful awareness.

The limitations of the present concern both the available evidence base and the methodological scope of this scoping review. This scoping review is subject to several limitations that reflect both the early stage of the field and the methodological challenges of synthesizing a small and heterogeneous body of evidence. Only ten studies met the inclusion criteria, highlighting the limited empirical base for dance-based mindfulness in non-clinical, recreational, and educational settings. Many seemingly relevant works were excluded due to their clinical orientation, absence of dance-specific protocols, or purely conceptual framing, highlighting the definitional ambiguity around what qualifies as a “dance-based mindfulness” intervention.

The included studies varied widely in design (from RCTs to case studies), duration (from single sessions to year-long programs), and methodological rigor. Few employed fidelity checks, standardized outcome measures, or longitudinal follow-ups, and most relied heavily on self-report data, with limited use of physiological or behavioral assessments. This heterogeneity restricts comparability and weakens conclusions about efficacy and sustainability.

Another key limitation is the cultural narrowness of the evidence base. Research has been concentrated in European, North American, and Australian contexts. Several interventions drew on traditional forms such as Sufi dance, or Taiji without engaging with their cultural or historical contexts, raising concerns about appropriation and decontextualization. Few studies examined participants’ cultural identities, social positions, or community contexts, leaving questions about inclusivity, accessibility, and the ways mindful dance might reinforce or challenge cultural narratives of embodiment.

Future research should establish clearer conceptual boundaries distinguishing dance-based mindfulness from general mindful movement or somatic training. Methodological rigor can be improved through standardized manuals, fidelity checks, multi-method outcome assessments, and long-term follow-ups. Equally important is expanding the cultural scope of research through participatory and community-led approaches that embed interventions within local traditions and values. Embedding programs within educational, recreational, and community infrastructures, rather than offering them as short-term adjuncts, will also be critical for ensuring continuity and broader impact.

Another limitation relates to self-selection bias. Most participants volunteered for dance-based mindfulness programs, likely reflecting a pre-existing interest in dance or movement practices. As a result, some may have entered interventions with already high levels of well-being or emotion regulation, limiting the scope for observable change. Not all studies compared pre–post outcomes with normative data, which constrains conclusions about intervention effects. Future work should address this by recruiting more diverse samples and embedding interventions in different universal settings.

These limitations illustrate both the promise and the challenges of an emerging field. Mindfulness-based dance holds potential as a culturally responsive and somatically rich approach to well-being, but its realization depends on greater theoretical clarity, methodological innovation, and cultural inclusivity.

## 5. Conclusions

This scoping review highlighted the conceptual promise and practical underdevelopment of dance-based mindfulness interventions in non-clinical, amateur and recreational contexts. Despite the growing academic and applied interest in embodied mindfulness, only a small number of empirical studies met the inclusion criteria. This limited evidence base reflects broader methodological and epistemological challenges, including varying definitions of mindfulness, partial integration of dance-specific principles, and a tendency to frame interventions as therapeutic or extracurricular rather than as embedded pedagogical practices.

At the same time, the included studies suggest that mindful movement practices, whether through Sufi-inspired dance, improvisational DMT, or somatic-based programs, can foster mindfulness-related traits such as emotional regulation, body awareness, and compassion, while supporting broader well-being. These benefits appear particularly relevant when interventions are culturally grounded, somatically rich, and socially meaningful.

The findings indicate that dance-based mindfulness remains an emergent but promising area of study. To move the field forward, future research should focus on developing theoretically grounded, culturally responsive, and pedagogically embedded interventions, supported by rigorous and diverse methodological approaches. Such work will be critical for realizing the full potential of dance-based mindfulness as a resource for individual well-being and community health.

## Figures and Tables

**Table 1:** Application of the SPIDER framework in this scoping review.

SPIDER	Description	Application in this Review
S—Sample	Who is being studied	Non-professional, non-clinical, amateur, recreational, and community dancers (children, adolescents, adults)
PI—Phenomenon of Interest	What is being studied	Dance-based mindfulness interventions integrating mindfulness principles with dance or expressive movement
D—Design	Study types considered	Intervention studies, protocols, and program evaluations
E—Evaluation	Outcomes of interest	Psychological, emotional, cognitive, physical, and social outcomes, where reported (e.g., stress reduction, emotional regulation, interoceptive awareness, balance, social connectedness).

**Table 2:** Search strategy results across databases.

Database	Total Records	Included Studies	Observations/Notes
PubMed	77	6	Found general studies on dance and mindfulness in schools and universities; few non-clinical recreational settings.
ERIC	89	8	Several studies on mindfulness-based movement; 8 relevant to amateur/recreational populations.
Scopus	69	6	Most studies involved clinical populations and settings
Web of Science	72	5	Most studies involved clinical populations and professional settings; a few relevant to community dance
PsycINFO	64	7	Most studies involved clinical populations; 6 relevant to non-clinical recreational dancers.
Google Scholar	131	9	Supplementary tool; mixed results and grey literature. Two phenomenological studies.

**Table 3:** Overview of included studies by design, source, and country.

Study	Design Type	Source	Country
Duberg et al. [60]	Quantitative—RCT	Peer-reviewed	Sweden
Hagensen [65]	Qualitative and Quantitative—Case Study	Peer-reviewed	USA
Marich & Howell [32]	Qualitative –Phenomenological	Peer-reviewed	USA
Pizarro et al. [61]	Quantitative—Quasi-experimental	Peer-reviewed	Spain
Rodríguez-Jiménez et al. [36]	Quantitative—RCT (Mixed-Methods)	Peer-reviewed	Spain
Shim et al. [63]	Quantitative—Mixed-methods, non-randomized one-group pre/post	Peer-reviewed	USA
Theroux et al. [66]	Quantitative—Single-arm pilot pre/post	Peer-reviewed	Australia
Areskoug Sandberg [41]	Mixed-methods	Grey literature—PhD thesis	Sweden
Saum [62]	Mixed-methods	Grey literature—Undergraduate thesis	USA
Warner [64]	Mixed-methods	Grey literature—Master’s thesis	USA

RCT = Randomized Controlled Trial.

**Table 4:** Summary of included studies on dance-based mindfulness interventions.

Author	Participants	Dance Type	Duration	Mindful Techniques	Assessment Tools	Key Findings
Duberg et al. [60]	112 adolescent girls (13–18 years old) with self-reported internalizing problems (stress, psychosomatic symptoms), Sweden	African dance, jazz, contemporary (with improvisation)	8 months, twice/week, 75 min/session (moderate to vigorous intensity)	Relaxation, light massage in pairs, reflection	Self-rated health (5-point scale), graphic rating scale for dance experience	Dance group showed greater improvement in self-rated health than control at all follow-ups; effects sustained up to 12 months post-intervention. High adherence (67% ≥ 50% attendance) and very positive experience (91%).
Hagensen [65]	one early adolescent girl aged 11 years, USA	Dance/Movement Therapy-based holistic wellness curriculum (nutrition, mindfulness, movement, body image, friendships)	6 weeks curriculum + check-ins over 4 months; 9 sessions total	Mindful eating, breathwork (“five-point breath”), body awareness exercises, movement-based reflection	Youth Quality of Life–Research Version (YQOL-R), parent surveys, session transcripts	Overall Quality of Life score increased from 86.58 to 88.65, with the Self domain rising ~7 points; gains in comfort with appearance, sexual feelings, stress level, and communication with friends. Enjoyment and engagement were highest in movement-based activities (Identity Dance, games, props). Parents reported greater self-appreciation; DMT found feasible, enjoyable, and effective for supporting self-awareness and wellness.
Marich & Howell [32]	10 Caucasian females aged 18–61, with trauma histories, from a community in northeastern Ohio	Dancing Mindfulness (community-based, not formal DMT)		Integration of mindfulness principles into free-form dance; body scans; breath awareness; present-moment focus; non-judgmental awareness	Semi-structured interviews; thematic analysis	Reported improvements in body awareness, emotional expression, and self-compassion; facilitated trauma processing; promoted safety, empowerment, and emotional regulation in line with ISTSS trauma-informed practice principles
Pizarro et al. [61]	111 students (53 intervention, 58 control) aged 18–34 from a university in Spain	SG-MBI mindful dancing (group-based mindful movement protocol with music, based on Sufi dance principles)	5 weeks, 2 sessions/week, ~45 min each	Guided mindfulness with music; body awareness; breath–movement–music synchronization; group synchrony; present-moment attention; Karunesh Heart Chakra Meditation sequence; closing relaxation circle	Compassion for Others Scale—Spanish-validated version (CFO-S) and brief CFO-S (intervention group only); Identity Fusion (with classmates and people in general); Pemberton Happiness Index—vitality item; Level of Involvement scale; Shared Flow Short Form; Perceived Emotional Synchrony Short Form	Significant improvement in mindfulness dimension of compassion vs. control (maintained at 1-week follow-up); brief compassion measure increased immediately post-intervention; Kindness and Common Humanity mediated by shared flow; Common Humanity also mediated by perceived emotional synchrony; increased fusion with people in general but not class group; no change in total compassion, SWB, or other compassion dimensions.
Rodríguez-Jiménez et al. [36]	37 engineering students (18–26 years; 19 experimental, 18 control), from Universidad Europea of Madrid, Spain	Dance Movement Therapy (DMT) (creative movement, Laban-based)	10 sessions, 2 sessions/week, over 5 weeks, 90 min each	Body awareness work, somatic exploration, Hatha yoga warm-up, Laban movement qualities, kinesthetic empathy, group reflection, relaxation	BAQ, Scale of Body Connection Scale of Body Connection (SBCBA, SBCBD), SWLS, PSS, WHO-5, TECA), Salivary cortisol (ELISA), HRV (Firstbeat Bodyguard 2), D2 Test of Attention, RP30 Problem-Solving Test, Reflective diaries.	Significant increase in body awareness (BAQ, SBCBA), decrease in bodily dissociation (SBCBD), increase in life satisfaction (SWLS), increase in well-being (WHO-5), decrease in perceived stress (PSS), stable low cortisol in DMT vs. increase in control, increase in problem-solving (RP30) and attention (D2) in both groups (no group difference), no significant change in empathy (TECA), no clear HRV changes. Themes of embodiment, relaxation, playfulness, emotional expression, self-awareness, skill development, group trust, and meaning-making. Preventive effect on stress increase during exam period.
Shim et al. [63]	16 older adults (70.9 ± 4.2 years), with mild age-related cognitive decline from 15 states across both urban and rural regions in USA	Mindfulness-based Dance/Movement Therapy (M-DMT)	12 weeks, 60 min/week, online via Zoom	Body awareness, attention/focus, emotional health, sensory enrichment, interpersonal connection, memory and nostalgia, resilience cultivation; structured and free-style movement; group discussions; sensory props; daily mindful movement practice videos	UCLA Loneliness Scale; PROMIS Depression SF-4a; PROMIS Positive Affect SF; General Well-Being Schedule; UCLA Physical Activity Scale; Treatment Credibility/Expectancy Questionnaire; Satisfaction survey; Patient Global Impression of Change; semi-structured interviews	There were significant reductions in loneliness and depression and increases in positive affect and psychological well-being. Physical activity increased but the change was not statistically significant. Qualitative themes included improved social connection, enhanced quality of life, greater body awareness, and better coping skills. The online format facilitated safe engagement.
Theroux et al. [66]	133 students (mean age 6.61 years), from primary schools in Southeast Queensland, Australia	Mindful Movement Program (MMP) with dance and motor activities	19 school weeks in two terms	Breath awareness, body scan, emotional awareness, acceptance, non-judgmental attention integrated into dance activities	BRIEF-2 (Teacher Form); SDQ (Teacher report); BOTMP-2 (Brief Form and Body Coordination subscale); daily fidelity checklist; semi-structured teacher interviews; demographic data	Feasibility criteria met: high recruitment (91%) and retention (98%), high acceptability, moderate fidelity. Significant improvements in executive function (GEC −2.95), externalizing behavior, internalizing behavior and motor skills. Teachers reported strong integration into curriculum, high student engagement, and resource usability despite COVID-19 disruptions.
Areskoug Sandberg [41]	112 adolescent girls aged 13–18 with stress-related/internalizing problems, frequent school nurse visits for somatic or emotional symptoms from Sweden.	Mixed styles: street dance, show/jazz, contemporary, with improvisation and emotional themes	over 24 weeks, twice weekly, 75 min/session (15 min warm-up, 40 min dance, 15 min relaxation, 5 min reflection)	Relaxation (10 min), light massage, focus on enjoyment, acceptance, social inclusion (non-competitive)	Self-reported questionnaires on daytime tiredness, alertness, sleep, and school satisfaction (baseline, 8, 12, 20 months)	Significant reduction in daytime tiredness at all follow-ups; significant increase in alertness at 12 and 20 months (unadjusted); effects not significant after adjustment for baseline differences; high adherence, no adverse events reported
Saum [62]	7 university-level ballet students (Ballet II–IV), USA	Ballet (as part of curriculum) with added yoga intervention	7 Vinyasa yoga sessions over ~2 months	Vinyasa-style yoga integrated with breath awareness, mindful movement, and instructor-led reflection	Five Facet Mindfulness Questionnaire (FFMQ) at baseline, mid, and post; guided journals; instructor observation notes	Increase in total mindfulness scores on FFMQ from baseline to post-intervention; qualitative themes indicated greater body awareness, reduced performance anxiety, improved focus; high adherence, no adverse events reported
Warner [64]	High school students (grades 10–12), ages 15–18, enrolled in Intermediate Dance (beginner/intermediate) and Dance Company (advanced), USA	Dance-based movement program integrating multiple dance styles with mindfulness incorporated into improvisational and choreographic work	10 weeks (2 weeks introduction + 8 weeks mindfulness-based rehearsal process, twice a week for 90 min)	Breath awareness, 4-7-8 breathing, STOP practice, hand tracing, body scan, mindful walking, mindful coloring, senses meditation, somatic yoga; connection of mindfulness practices to improvisational movement creation and choreography	Questionnaire (baseline), 4 self-reflections (over 8 weeks), post-performance interviews; qualitative thematic analysis and quantitative summary of selected responses	Mindfulness curriculum was feasible and engaging for both beginner and advanced high school dancers; participants reported enhanced self-awareness, self-acceptance, emotional regulation, and creative connection between mindfulness and movement; student-led mindfulness practices emerged in advanced group; mindfulness supported social-emotional learning in dance education.

BAQ = Body Awareness Questionnaire, BOTMP-2 = Bruininks-Oseretsky Test of Motor Proficiency, Second Edition, CFO-S = Compassion for Others Scale (Spanish-validated version), D2 = D2 Test of Attention, DMT = Dance/Movement Therapy, ELISA = Enzyme-Linked Immunosorbent Assay, FFMQ = Five Facet Mindfulness Questionnaire, GEC = Global Executive Composite (from BRIEF-2), HRV = Heart Rate Variability, ISTSS = International Society for Traumatic Stress Studies, M-DMT = Mindfulness-based Dance/Movement Therapy, MMP = Mindful Movement Program, PROMIS = Patient-Reported Outcomes Measurement Information System, PSS = Perceived Stress Scale, RP30 = Problem-Solving Test (30-item), SDQ = Strengths and Difficulties Questionnaire, SBCBA = Scale of Body Connection—Body Awareness, SBCBD = Scale of Body Connection—Bodily Dissociation, SG-MBI = Sufi Group-based Mindful Bodywork Intervention (mindful dancing protocol), SWLS = Satisfaction with Life Scale, TECA = Test of Cognitive and Affective Empathy, WHO-5 = World Health Organization–5 Well-Being Index, YQOL-R = Youth Quality of Life—Research Version.

## Data Availability

No new data were created or analyzed in this study.

## Abbreviations

The following abbreviations are used in this manuscript:

BAQ	Body Awareness Questionnaire
BOTMP-2	Bruininks-Oseretsky Test of Motor Proficiency, Second Edition
CFO-S	Compassion for Others Scale (Spanish-validated version)
D2	D2 Test of Attention
DMT	Dance/Movement Therapy
ELISA	Enzyme-Linked Immunosorbent Assay
FFMQ	Five Facet Mindfulness Questionnaire
GEC	Global Executive Composite (from BRIEF-2)
HRV	Heart Rate Variability
ISTSS	International Society for Traumatic Stress Studies
M-DMT	Mindfulness-based Dance/Movement Therapy
MMP	Mindful Movement Program
PROMIS	Patient-Reported Outcomes Measurement Information System
PSS	Perceived Stress Scale
RP30	Problem-Solving Test (30-item)
SDQ	Strengths and Difficulties Questionnaire
SBCBA	Scale of Body Connection—Body Awareness
SBCBD	Scale of Body Connection—Bodily Dissociation
SG-MBI	Sufi Group-based Mindful Bodywork Intervention (mindful dancing protocol)
SWLS	Satisfaction with Life Scale
TECA	Test of Cognitive and Affective Empathy
WHO-5	World Health Organization–5 Well-Being Index
YQOL-R	Youth Quality of Life—Research Version

## Appendix A

Overview of the core components, rationale, delivery format, and implementation features of the included dance-based mindfulness interventions, based on the TIDieR checklist (Table A1).

**Table A1:** Summary of intervention characteristics based on the TIDieR framework.

**TIDieR Item**	**Description**	**Studies Reporting This**
Intervention (What)	Mindful dance/movement practices integrated with relaxation, reflection, body scans, breath awareness, yoga, sensory props, compassion-focused exercises, improvisation, emotional expression. Dance types included: African dance, jazz, contemporary, ballet, Sufi-inspired movement, street/show dance, mindful movement with motor activities, online M-DMT.	Duberg et al. [60]; Hagensen [65]; Marich & Howell [32]; Pizarro et al. [61]; Rodríguez-Jiménez et al. [36]; Shim et al. [63]; Theroux et al. [66]; Areskoug Sandberg [41]; Saum [62]; Warner [64]
Why (Rationale)	Based on mindfulness theory, DMT principles, somatic psychology, trauma-informed practice, embodied self-regulation, creativity and social-emotional learning in education, preventive health for stress-related problems, and promotion of resilience and well-being.	All studies
Who (Providers)	Licensed/certified dance/movement therapists, psychologists, health educators, yoga instructors, dance teachers/instructors, school teachers, or trained facilitators. In some community-based programs, provider credentials were not fully specified.	All studies (credentials vary)
What (Materials)	Meditation/relaxation music, reflection journals, yoga mats, props (scarves, objects), mindfulness scripts, standardized scales/questionnaires, salivary cortisol and HRV monitors, online video resources (for M-DMT), choreography assignments.	All studies (with variable specificity)
How (Mode of Delivery)	Primarily in-person group sessions; one intervention [63] delivered fully online via Zoom with daily video resources; school-based interventions integrated into curriculum.	All studies
Where (Setting)	Schools, universities, community centers, academic wellness programs, dance studios, online platforms.	All studies
How much (Dosage)	Ranged from short-term programs (5 weeks, 2 sessions/week) to long-term (24 weeks, twice weekly; 8 months, twice weekly). Session length varied (40–90 min). Online M-DMT: 12 weeks, weekly. School-based MMP: 19 weeks.	All studies
Tailoring (Modifications)	Some programs tailored to participant needs (e.g., reflection, trauma-informed care, adaptive movements for age/cognitive decline). Explicit mention of tailoring found in Rodríguez-Jiménez [36], Shim [63], Warner [64].	Subset of studies
How well (Planned Fidelity)	Some reported fidelity measures (Theroux [66]: fidelity checklist; Pizarro [61]: structured protocol; Rodríguez-Jiménez [36]: reflective diaries; Duberg [60] & Areskoug [41]: high adherence tracking). Others less explicit.	Reported in multiple studies, but variable detail
How well (Actual Adherence/Engagement)	High adherence reported in many studies Duberg et al. (Duberg et al., 2013) 67% ≥ 50% attendance; Sandberg [41]: high adherence, no adverse events; Theroux et al. [66]: 91% recruitment, 98% retention; Saum [62] & Shim et a;. [63]: high adherence and satisfaction). Enjoyment and positive engagement consistently reported.	All studies

## Appendix B

Overview of the distribution of studies by age group, highlighting both the most frequently examined populations and those less represented in the literature (Table A2).

**Table A2:** Age groups of participants in included mindfulness-based dance interventions.

**Age Group**	**Studies**	**Participants**
Primary school children (≤12 yrs)	Theroux et al. [66]	133 primary school students (mean age 6.61 years)
Older children/early adolescent	Hagensen [65]	1 early adolescent girl, 11 years old
Adolescents (13–18 yrs)	Duberg et al. [60]; Areskoug Sandberg [41]; Warner [64]	112 adolescent girls aged 13–18 (two studies), high school students aged 15–18
Young adults (18–40 yrs)	Pizarro et al. [61]; Rodríguez-Jiménez et al. [36]; Saum [62]	University students and early-career adults aged 18–40
Adults (mixed age, 18–80 yrs)	Marich & Howell [32]	Adults aged 18–61 (trauma sample)
Older adults (≥65 yrs)	Shim et al. [63]	16 older adults (mean age 70.9 years) with mild cognitive decline
